# Exploring the Potential of Cannabinoid Nanodelivery Systems for CNS Disorders

**DOI:** 10.3390/pharmaceutics15010204

**Published:** 2023-01-06

**Authors:** Mariana Kolesarova, Patrik Simko, Nicol Urbanska, Terezia Kiskova

**Affiliations:** Institute of Biology and Ecology, Faculty of Science, Pavol Jozef Safarik University in Kosice, 040 01 Kosice, Slovakia

**Keywords:** nanoparticles, lipids, cannabinoids, THC, CBD, neurodegenerative diseases, targeted delivery, endocannabinoid system, brain

## Abstract

Cannabinoids have a major therapeutic value in a variety of disorders. The concepts of cannabinoids are difficult to develop, but they can be used and are advantageous for a number of diseases that are not sufficiently managed by existing treatments. Nanoconjugation and encapsulation techniques have been shown to be effective in improving the delivery and the therapeutic effectiveness of drugs that are poorly soluble in water. Because the bioavailability of cannabinoids is low, the challenge is to explore different administration methods to improve their effectiveness. Because cannabinoids cross the blood-brain-barrier (BBB), they modify the negative effects of inflammatory processes on the BBB and may be a key factor in the improvement of BBB function after ischemic disease or other conditions. This review discusses various types of cannabinoid administration, as well as nanotechnologies used to improve the bioavailability of these compounds in CNS diseases.

## 1. Introduction

*Cannabis sativa* L. is one of a variety of plants that have been used for a thousand years in agriculture, textiles and medicine, among many societies [1,2]. As industrial hemp is similar to the medicinal type of *Cannabis*, its production has been banned for several years, which has destroyed centuries of educational and genetic materials [3]. In the past 20 years, most countries have legalized industrial hemp production, leading to significant research on the health benefits of hemp products and hemp-derived products [1,2]. Concomitantly, the last few decades have provided new insights into the therapeutic potential of cannabinoids in human health.

More than 500 cannabinoids have been discovered in *Cannabis sativa* L., including phenolic compounds, steroids, cannabinoids, terpenoids, fatty acids and hydrocarbons [4,5]. Terpenoids, amides, oxylipins and amines create the typical aroma of *Cannabis* [6,7]. Tetrahydrocannabinol (THC) was first isolated by Gaoni and Mechoulam in 1964 [8]. Based on the concentration of the main cannabinoids (THC, cannabigerol and cannabidiol (CBD)), *Cannabis* is divided into five chemotypes: (a) drug-type plants with a high amount of THC, (b) *Cannabis* in medicine—the content of THC/CBD is 1/1, (c) industrial fiber *Cannabis* with a minimal content of THC and a significant amount of CBD, (d) fiber-type hemp with CBD content, and (e) fiber-type plants without cannabinoids [9]. Products with psychoactive effects include marijuana, hashish oil or hashish. Marijuana, which is a mixture of dried leaves and female inflorescences, contains approximately 2–6% of THC. Hashish oil contains 50% of THC and is produced from an extract or a resin. The THC content in hashish is approximately 12%, and it is produced from resin (which protects the tops of female plants) [10]. With regards to the dose, the effects of THC include anxiety, memory impairment, and psychotic symptoms, and CBD balances these negative effects of THC [11,12]. The best-known cannabinoids are THC and CBD, both of which are characterized by low solubility in water but high solubility in most solvents (such as alcohol or lipids) [4].

Due to the process of legalization, the use of marijuana in the last year of young adults aged 19–30 years increased significantly, compared with five and ten years earlier, reaching historical highs [13,14,15]. Concomitantly with the increased use of cannabinoid, rapid assays for on-site cannabinoids detection in oral fluids have been developed [16,17].

However, the use of cannabis is associated with various health risks [18]. Generally, the use of THC is linked to seizures, respiratory depression, and cardiovascular complications [19]. CBD has long been considered a risk-free compound. However, in animals, the adverse effects of CBD include developmental toxicity, embryo-fetal mortality, neurotoxicity, hepatocellular injuries, spermatogenesis reduction, organ weight alterations, male reproductive system alterations, and hypotension, as previously reviewed [20]. Human CBD studies on epilepsy and psychiatric disorders have reported CBD-induced drug-drug interactions, hepatic abnormalities, diarrhea, fatigue, vomiting, and somnolence [21]. A case report referred to toxicity from CBD gummy ingestion [22].

Once CBD or THC enters the body, it is metabolized in the liver and intestine, where cytochrome enzymes and glucuronyltransferases produce hydroxylated and carboxylated metabolites [20,23]. Cannabinoids administered via inhalation exhibit pharmacokinetics similar to those of administered intravenously [24]. Cannabinoids are distributed into well-vascularized organs such as the heart, liver, brain, and lungs, with subsequent equilibration into organs such as the spleen, adipose tissue, and renal tissue [23,25].

### 1.1. The Endocannabinoid System in the Brain and Cannabinoid Receptors

The Central Nervous System (CNS) is a complex entity in living organisms, and in the treatment of neurodegenerative disorders, it is necessary to tackle the most significant challenges, such as cross-border crossing of the blood-brain barrier (BBB). Currently, the treatment of CNS diseases is mainly symptomatic with no disease-modifying therapies for most disorders [26].

The endocannabinoid system (ECS) is a complex system involved in many physiological processes in mammals (homeostasis, anxiety, feeding behavior/appetite, emotional behavior, neural function, neurogenesis, neuroprotection, pain perception, fertility, pregnancy, and pre- and postnatal development), as well as some pathological processes (depression) [27,28,29]. Moreover, ECS affects several cognitive and neurophysiological processes such as motor function, memory, learning, energy metabolism, inflammation, nociception, and neuroprotection [27,30].

The components of the ECS include (1) receptors, (2) their ligands, and (3) enzymes, responsible for the biosynthesis and degradation/deactivation of ligands [31]:

(1) the receptors with which cannabinoids interact can be divided into three main classes or groups: (i) G protein-coupled receptors (GPCRs) (e.g., cannabinoid type 1 receptor (CBr1) and cannabinoid type 2 receptor (CBr2)) [31], (ii) ligand-sensitive ion channels (e.g., transient receptor potential vanilloid 1, TRPV1). TRPV1 is also activated by various chemicals, physical stimuli, capsaicin and ions, and (iii) nuclear receptors (e.g., PPARs) [32,33].

These receptors are present in both the CNS and the periphery. Downstream signalling of these CBrs is significantly involved in variety of standard functions, as well as in several pathophysiological functions of the CNS [31].

**Figure 1:**
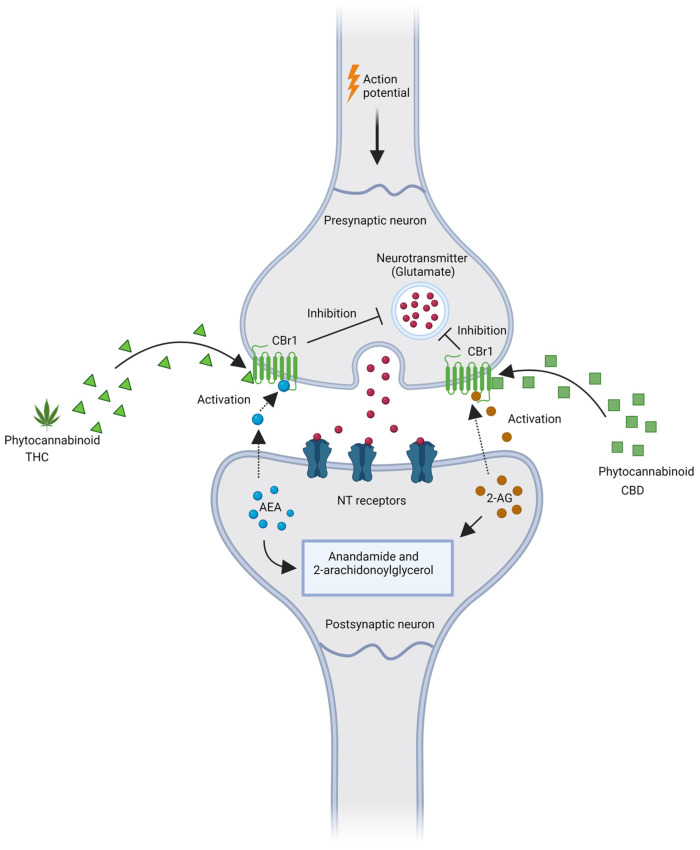
Simplified scheme of the basic modulation of the endocannabinoid system by phytocannabinoids (CBD and THC). The main mechanism by which endocannabinoids regulate synaptic function is retrograde signaling [37]. Once released from the postsynaptic neuron, endocannabinoids bind to CBr1 located on the presynaptic membrane to inhibit the release of neurotransmitters. Endocannabinoids are removed from the synaptic junction after CBr1 activation via cellular transport followed by hydrolysis. AEA is hydrolyzed in postsynaptic neurons by fatty acid amide hydrolase (FAAH), terminating its action. After CBr1 activation, 2-AG is hydrolyzed in presynaptic neurons by monoacylglycerol lipase (MAGL). This retrograde signaling provides an inhibitory feedback mechanism to regulate neurotransmitter release in the brain. [38,39]. NT- neurotransmitter receptor. Created with BioRender.com, accessed on 28 November 2022.

(2) endogenous ligands, the most studied of which are anandamide (N-arachidonoyl ethanolamine, AEA) and 2-arachidonoylglycerol (2-AG) (Figure 1);
(3) endocannabinoid metabolic enzymes are responsible for the synthesis and degradation of endocannabinoids: (i) N-acylphosphatidylethanolamine (NAPE)-specific phospholipase D-like hydrolase (NAPE-PLD), which catalyzes the synthesis of AEA and other N-acylethanolamines [34], and fatty acid amide hydrolase (FAAH), which catalyzes the hydrolysis of AEA (and other N-acylethanolamines and primary fatty acid amides) [31]. (ii) Diacylglycerol lipase α (DAGLα) and DAGLβ catalyze the biosynthesis of 2-AG and other monoacylglycerols [35] and monoacylglycerol lipase (MAGL) catalyzes the hydrolysis of 2-AG (and other monoacylglycerols) [36].

Both AEA and 2-AG are lipophilic and are synthesized on demand from membrane phospholipids, whereby they can readily partition into and diffuse across cell membranes without being stored in vesicles. The body-specific molecules that interact with CBRs to influence biological processes are referred to as endocannabinoids.

However, there is also evidence suggesting that endocannabinoid signalling occurs in a non-retrograde or autocrine manner, where it can modulate neural function and synaptic transmission by engaging the transient receptor potential TRPV1 and CBr1s located on or within the postsynaptic cell. This non-retrograde signaling regulates self-inhibition via CBr1- and CBr2 receptor-dependent reductions in excitability and synaptic plasticity via a TRPV1-mediated form of long-term depression [40].

### 1.2. The Blood-Brain-Barrier and Cannabinoids

**Figure 2:**
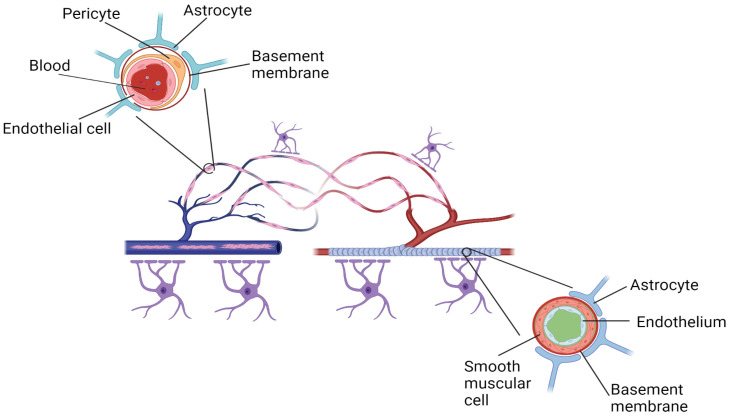
The neurovascular unit (upper part of the image) represents an interactive network of vascular cells (pericytes and endothelial cells), glia (astrocytes and microglia), and neurons. The blood-brain barrier is centrally positioned within the neurovascular unit and is formed by a monolayer of endothelial cells extending along the vascular tree. Created with BioRender.com, accessed on 28 November 2022.

The blood-brain barrier (BBB) is an entity with multiple aspects, including metabolism, transport, and structural components [41]. It is an endothelial membrane, that, together with the neurovascular unit (NVU, Figure 2), restricts the entry of toxins, pathogenic organisms, and blood cells into the brain [42]. The NVU is a minimal functional unit of the brain that is composed of vascular cells, glial cells, and neurons. It maintains BBB integrity and controls the supply of cerebral blood flow, both of which are key to maintaining normal brain function [43].

The BBB represents a strict control of what enters the brain and is formed by tight junctions between the endothelial lining blood vessels, astrocyte endfoot, and a basement membrane. The ability of a drug to cross the BBB should be considered if administered at the peripheral level, but its target is located in the brain [44].

Breakdown/dysfunction of the BBB has been associated with several neurodegenerative disorders such as Alzheimer’s disease (AD) [45], amyotrophic lateral sclerosis (ALS) [46], Parkinson’s disease (PD) [47], multiple sclerosis [48], chronic traumatic encephalopathy [49], or stroke [50], epilepsy [51] and many others.

Activation of the ECS plays a role in protecting the interactions between immune and endothelial cells and in neuroprotection by maintaining tight junctions in the BBB [52]. The BBB is sensitive to the consequences of chronic systemic inflammation as it leads to brain inflammation and consequently impairs its integrity. For example, CBD has been identified to modulate endothelial and epithelial barriers and to exhibit immunosuppressive activity, improving the deficits in the cognitive system [53]. In a model of multiple sclerosis, CBD reduced the crossing of leukocytes through the BBB from the systemic circulation by microglial attenuation and modulation of chemokine expression [54]. At doses of 5, 10 and 20 mg/kg, CBD decreased the number of aquaporin-4-positive and glial fibrillary acidic protein-positive cells when administered intraperitoneally. Concomitantly, it reduced the protein levels of some pro-inflammatory cytokines and increased the expression of claudin-5 and occludin, which are tight junction proteins. CBD administration improved the integrity and permeability of the BBB and reduced edema in the brain after traumatic brain injury [55]. Cannabis also had a beneficial effect on HIV-associated BBB injury. Since BBB disruption may permit increased entry of toxins, such as microbial antigens and inflammatory mediators, with consequent CNS injury, these results support a potential therapeutic role of cannabis among patients with HIV-virus [56]. Schou et al. (1977) sowed that THC efficiently crosses the BBB [57] and placental barriers, and thus can be found in the milk of breastfeeding mothers [58].

The integrated defence systems of the BBB impose a major challenge for effective drug delivery and the treatment of many brain diseases. Over the past decade, multiple strategies to improve drug delivery across the BBB have focused on non-invasive techniques [59].

### 1.3. Drugs Based on CBs from Cannabis sativa

To date, the Food and Drug Administration (FDA) has approved one drug product derived from *Cannabis*, Epidiolex (CBD), and three synthetic drug products, namely, Marinol (dronabinoid), Syndros (dronabinoid) and Cesamet (nabilone). These approved drugs are only available with the prescription of a registered healthcare provider (https://www.fda.gov/news-events/public-health-focus/fda-and-cannabis-research-and-drug-approval-process, accessed on 28 November 2022).

Nabilone (Cesamet^®^) and Dronabinol (Marinol^®^) are synthetic molecules that mimic the pharmacological activity of THC [60].

Dronabinol is a synthetic tetrahydrocannabinol (THC), which was approved by the FDA in 1985 for the treatment of HIV/AIDs-induced anorexia and chemotherapy-induced nausea and vomiting in patients who have failed to respond to conventional antiemetics (Table 1). Dronabinol has also been used off-label for the treatment of obstructive sleep apnea. This activity provides an overview of the pharmacology of dronabinol, its indications and usage, adverse effects, contraindications, and other pertinent information [61].

Nabiximols (Sativex^®^) was first approved as a botanical drug in the UK in 2010. The aerosol mouth spray contains an extract from the *Cannabis* plant and flowers derived from two *Cannabis* plant varieties. It contains nearly equal amounts of THC and CBD but also minor quantities of cannabinoids and other compounds from the plant [60].

These drugs have several side effects, such as seizures, fast heartbeats, light-headedness, confusion, sleeping problems, memory problems, difficulty concentrating, unexpected changes in mood, slurred speech, stomach pain, severe headache, blurred vision, anxiety, and nosebleed [20,61,62,63].

Clinical trials have started using various cannabinoids (see the list in [64]).

**Table 1:** Cannabinoids-based drugs approved by FDA for medical use.

Drug Name	Active Ingredient	Compound	Medicinal Use	References
Marinol	Dronabinol	THC	symptoms of nausea and vomiting after chemotherapy appetite loss caused by AIDS	[61]
Syndros	Dronabinol	THC	symptoms of nausea and vomiting after chemotherapy appetite loss caused by AIDS	[61]
Cesamet	Nabilone	THC	symptoms of nausea and vomiting after chemotherapy	[63]
Epidiolex	Cannabidiol	CBD	Dravet syndrome Lennox-Gastaut syndrome	[20,62]
Sativex	Nabiximols	CBD THC	neuropatic pain and symptomatic relief of spasticity during multiple sclerosis	[65]

## 2. Strategy and Source of Data Collection

The search of publications of interest was conducted by questioning PubMed, Web of Science and google search databases. Each database was searched from the emergence date until 30 November 2022. A combination of specific vocabulary terms and free-text terms relating to cannabinoids and nanosystems were included. The databases were questioned for “cannabinoids + nanosystems” “cannabinoids + nanoparticles” “cannabinoids + drug delivery systems”. Duplicates of the databases search were excluded. Study inclusion criteria included peer-reviewed publication in English, case reports, reviews, or editorials.

## 3. Results

### 3.1. Cannabinoid Nanoparticles in CNS Diseases

CNS diseases represent a specific group of diseases that must solve the problem of effective drug delivery through the BBB. In this context, since the effective brain drug delivery should not rely on passive targeting, active targeting using nanomedicines has been intensively studied. In regard to this, the use of nanocarriers can be considered as an alternative to enhance the passage across the BBB [66]. Nanoformulations have resulted in high CBD solubility, encapsulation efficiency, stability, and sustained CBD release [67]. The advantage of the nanotechnology is the ability to deliver higher concentrations of drugs to the targeted area and to reduce the accumulation of drugs in peripheral areas. Several factors have been monitored during drug delivery, including the location of drug administration, rate of CSF production, lipophilicity, transition of the barrier, volume of drug distribution, its permeability, physicochemical properties and clearance rate [26]. When considering the distribution of nanoparticles, the main question is if they reach the brain. Many researchers hope that the studied drugs will enter the brain if nanosystems are used for drug delivery. Others are concerned about unintentional nanoparticle entry into the brain and possible adverse effects [44]. However, the use of nanomedicine still suffers from certain critical issues such as toxicity at high-dose levels, passivation due to multiple inorganics, and low pH sensitivity [68]. Primary toxicity effects include DNA damage induced by reactive oxygen species (ROS) production and inflammation. After potential unwanted redistribution to secondary organs and tissues, nanoparticles may alter cellular functions [69].

Cannabinoids have poor oral bioavailability (approximately 6% of the similar dose administered intravenously). Pure cannabinoids, such as CBD and THC, show similar plasma concentration-time profiles, with a delay of 120 min before reaching the peak concentration [24,70]. The bioavailability increases when co-administered with food lipids [64]. When cannabinoids are not metabolized in the liver, their bioavailability increases to 22% [71]. In contrast, when CBD was administered with high-fat meal, bioavailability increased from 6% to 25% [72].

Bioavailability following the smoking route was reported to be 2−56%, partly due to intra- and inter-subject variability in smoking dynamics, which contributes to uncertainty in dose delivery [73]. Orotransmucosal drug delivery is an alternative non-invasive administration route that avoids gastrointestinal decomposition and hepatic first-pass metabolism when achieving systemic drug circulation [74]. When smoking, lung availability ranged from 12% for mixed cannabis material with relatively low THC content to approximately 19–27% for marijuana flowers, similar to THC in marijuana and CBD in CBD-rich marijuana [75]. The plasma profiles of THC after smoking and intravenous injection were similar, whereas the plasma levels after oral intake were low and irregular, indicating slow and erratic absorption [76].

#### 3.1.1. Self-Emulsifying Drug Delivery System

Currently, some of the strategies used in product marketing are based on the use of salt formation (i.e., pH adjustment), co-solvency (e.g., ethanol and propylene glycol), micellization (e.g., polysorbate, cremophor oil), (nano)-(micro)-emulsification, complexation (e.g., cyclodextrins), or encapsulation in lipid-based formulations and nanoparticles [77]. For example, Esposito et al.(2016) developed and optimized a method to encapsulate potent and expensive cannabinoid drugs in nanostructured lipid carriers, namely, URB597, AM251 and rimonabant [78].

During the last five years, evidence regarding the use of nanoparticles in cannabinoids delivery has increased. CBD has a limited oral bioavailability. To overcome this limitation, Knaub et al. (2019) developed a novel self-emulsifying drug delivery system (SEDDS) based on VESIsorb^®^ formulation technology incorporating CBD [79]. SEDDS are mixtures of oils and surfactants that also contain hydrophilic solvents [80,81]. They undergo spontaneous emulsification after contact with gastric intestinal fluid and slight agitation, as is the case in the gastrointestinal tract [82,83]. Based on new studies on lipase activity, pH, and levels of bile salts in the gastrointestinal tract of healthy adults or young volunteers as well as in a population of non-healthy individuals, an understanding of the in vivo digestion of SEDDS has been enabled [84]. SEDDS-CBD showed a significant increase in CBD levels in blood plasma, increased bioavailability, and rapid absorption in healthy patients. Some sex-based differences have also been observed [79]. A formulation of PTL101 based on oral gelatin matrix pellets containing pure CBD embedded in seamless gelatin matrix beadlets was tested for safety and tolerability at two different single-administered concentrations of 10 and 100 mg of CBD. It was administered to healthy volunteers, and the bioavailability of CBD was compared with that of the Sativex spray. The bioavailability of CBD increased markedly compared to that of the spray. PTL101 is a user-friendly oral concept that shows safe and efficient delivery of CBD [85]. To date, various cannabis-based self-emulsifying product patents have been developed, showing that they will be effective in cannabinoid delivery [86,87,88,89,90].

#### 3.1.2. Lipid Nanoparticles

As cannabinoids are lipophilic compounds, the application of different stimulants and delivery systems to improve the solubility and bioavailiability of cannibinoids must be considered [91]. The physicochemical properties associated with poor long-term stability and psychoactive effects pose further challenges for the delivery of cannabinoids [77]. Lajoie et al. (2022), described the effect of various emulsifiers, specifically whey and soy protein isolate, as well as Tween 80, on the ability to encapsulate cannabis oil with maltodextrin. Their results suggest that the spray drying of nanoencapsulated cannabis oil using tested emulsifiers has a significant impact on its encapsulation effectiveness and bioavailability and highlights the importance of choosing the appropriate emulsifying agents for optimal oral administration [92].

#### 3.1.3. Lipid Components in Self-Emulsifying Drug Delivery System

To investigate the effects of lipid components in self-emulsifying drug delivery systems on the oral absorption of THC and CBD, a free-moving rat model was used. These results indicate that the effects of each lipid type on cannabinoid bioavailability cannot be easily predicted. The differences in the absorption effects of long-chain triglycerides and middle-chain triglycerides were not significant for the Type I formulations but were more prominent in the Type II formulations. Concomitantly with these results, an unpredictable in vivo behavior indicates the importance of pre-clinical testing of each vehicle, following in vitro investigations [93]. Another lipid-based drug delivery system was used to show time and concentration dependence after oral administration of CBD-sesame oil with prolonged drug input in comparison with the CBD-SEDDS. Moreover, it has been observed that less lipophilic compounds such as ibuprofen leave the stomach much earlier than lipophilic CBD in sesame oil, showing different absorption kinetics [94]. As hypothesized by the same research group, THC and CBD have poor absorption in the colon compared to the small intestine. The suggested formulation examined in vitro was a floating gastro-retentive tablet based on egg albumin matrix, gas-generating agents, and surfactants. In vivo investigation of CBD-containing formulations in a freely moving rat model showed a prolonged absorption phase with a substantial increase in bioavailability compared to the CBD solution [95]. Izgelov et al. (2020) investigated the oral absorption processes of synthetic CBD administered in different oral formulations (oils or oil-based solutions) in 12 healthy male volunteers. Administration of CBD in different lipid-based vehicles resulted in different absorption behaviors, concretely in two absorption behaviors of early and delayed absorption among subjects, as opposed to SEDDS platform that resulted in a uniform early absorption profile. The results of this study highlight the importance of the solubilization process of lipophilic drugs [96].

Specifically, in the field of neurodegenerative diseases, the role of cannabinoid-derived nanosystems may be of high importance, as they may not only diminish the symptoms, but also slow the process of the disease [97] (for more details see Table 2). Evidence suggests that cannabinoids may be therapeutically useful in dementia because they target several underlying pathophysiological processes linked to dementia [98]. Owing to the limited brain accumulation of therapeutics, nano- or micro-sized droplets of such formulations have gained significant importance [99]. The choice of the oily phase was based on the solubility of CBD, which helps achieve both high encapsulation efficiency and drug loading. Lipid nanocapsules are biocompatible and biodegradable carriers for CBD with a prolonged release platform. Moreover, the size of lipid nanoparticles plays a pivotal role in the extent of CBD release [100]. The aim of the study by Mihailova et al. (2022) was to evaluate the physicochemical and biopharmaceutical features of nanoliposomes and nanostructured lipid carriers loaded with *Cannabis sativa* extract intended for safe and efficient transport via the BBB and the treatment of epilepsy using male ICR mice [101]. The authors used nanoliposomes and nanostructured lipid formulations that were <200 nm in diameter. The prepared nanoparticles showed markedly higher antioxidant activity than that compared of the extract alone. In mice, during testing of the anticonvulsant activity, all formulations significantly elevated the latencies for myoclonic, clonic, and tonic seizures and, therefore, could be used to prevent different types of epilepsy seizures [101]. Amini and Abdolmaleki (2022) studied nano-chitosan in combination with CBD in Alzheimer’s disease in Wistar rats. They revealed positive behavioral changes in the Morris water maze test. Moreover, the protein expression levels of CBr1 and CBr2 increased significantly. CBD coated with nano-chitosan has good potential for reducing Aβ plaques and improving learning and memory in Alzheimer’s rats [102]. During neuropathic pain, CBD with nanostructured lipid carriers (NLC) (concrete particles with a positively charged surface, employing stearic acid, oleic acid, Span 20^Ⓡ^, and cetylpyridinium chloride) were tested. Both formulations, CBD-NLC and CBD-NLC-gel, showed high mucoadhesion in vitro. In vivo, CBD-NLC dispersion (without gel), administered intranasally, produced a more significant and lasting antinociceptive effect in animals with neuropathic pain than oral or nasal administration of CBD solution. However, nasal administration of CBD-NLC-gel did not decrease mechanical allodynia [103]. Aparicio-Blanco at al. (2019) revealed that the surface functionalization of these lipid nanocapsules with CBD allowed cannabinoid receptors overexpressed in glioma cells to be targeted, as shown in permeability experiments across the BBB of an in vitro model and in biodistribution experiments in mice [104]. When testing cannabinoid nanoparticles on other cancer models, the results showed enhancement of photodynamic therapy in combination with cannabinoids in colorectal [105], cervical [106] and breast cancer [107,108,109]. CBD in combination with nanomiceles was studied in triple-negative breast cancer [110], and with lipid nanoparticles in ovarian cancer [111]. The transferring (Tf) surface-modified 9-THC-loaded poly(lactide-co-glycolic) nanoparticles (Tf-THC-PLGA NPs) were evaluated as a highly promising approach for colorectal cancer [112].

### 3.2. Targeted Brain Delivery of Cannabinoid-Based Nanoparticles

As discussed before, the main question in the treatment of CNS diseases with cannabinoid-based nanostructured drugs is whether the nanoparticles reach the designated target location in the brain. For this reason, the so called dual- and multi-targeted nanoformulations are being developed. Dual- and multi-targeted nanoparticles integrate different targeting functionalities and have provided a paradigm for precise drug delivery to the exact pathological location in the brain [113].

In general, the optimization of nanoparticle delivery requires a design that fulfils several important conditions. It must overcome the BBB, specifically target the desired location, and trigger internalization by the target cells. Furthermore, enabling endo/lysosomal escape, navigation to the target organelle and controlled drug release is necessary [113,114].

In vitro drug release has been investigated in several studies. For example, CBD liquisolid powder prepared with volatile and nonvolatile solvents had a better CBD release performance than the CBD formed as the surfactant-based and control powders. In addition to drug release, liquid vehicles significantly influenced mucosal permeation and deposition, either enhanced or suppressed, in liquisolid systems [74]. In another study, CBD was encapsulated into nanoparticles with low polydispersity and high drug loading via Flash NanoPrecipitation, using hydroxypropyl methylcellulose acetate succinate and lecithin as amphiphilic particle stabilizers. These nanoformulations showed more rapid and complete in vitro dissolution kinetics than CBD alone, representing a 6-fold improvement in dissolution compared to crystalline CBD [115].

Until now, published works have focused mainly on the process of overcoming the BBB. However, a comprehensive description of targeting strategies for drug delivery to pathological sites, abnormal cells, and their subcellular compartments is still lacking.

When discussing brain cancer, targeting the tumor tissue was achieved using Tf-THC-PLGA NPs. Tf-THC PLGA NPs decreased cell viability to 17% in comparison with 88% of plain nanoparticles, despite their slower internalization rate. Nanoparticle internalization occurred through cholesterol-associated and clathrin-mediated mechanisms. Overall, Tf-modification of PLGA NPs is a highly promising approach for THC-based antitumor therapies, potentially maximizing the amount of drug released in a sustained manner on the surface of cells bearing cannabinoid receptors [112]. Another study by Aparicio Blanco et al. (2019) introduced a pilot brain tumor targeting strategy with CBD [116]. They found that small lipid nanocapsules loaded with CBD may be used as dual-target candidates for targeted intravenous treatment of gliomas [116]. However, it is appropriate to state that these dual nanosystems are relying on the fact, that the brain endothelium expresses the same receptors as brain cancer cells. Dual-ligand nanomedicines are being developed and evaluated not only for brain cancer, because this duality gives them versatile functions and has the potential to improve the efficacy of tumor-targeted delivery and cancer treatment [113,117]. 

**Table 2:** Some cannabinoids-based treatments for selected neurological disorders.

Disease	Compound	Dose	Model	Effect	Receptor Involvement	References
Parkinson’s disease	CBD	3 mg/kg weight	male Sprague-Dawley rats	recovery of 6-hydroxydopamine-induced dopamine depletion neuroprotective effects	CBr1 and CBr2 independent	[118]
		3 mg/kg weight	male Sprague-Dawley rats	waning of changes caused by 6-hydroxydopamine -induced dopamine depletion neuroprotective effects	CBr1 partly independent TRPV1	[119]
		75 mg and 300 mg/day	119 patients	motor and general symptoms impairment using higher dose of CBD	not listed	[120]
	THC	3 mg/kg weight	male Sprague-Dawley rats	waning of changes caused by 6-hydroxydopamine -induced dopamine depletion neuroprotective effects	CBr1 partly independent TRPV1	[119]
		2 mg/kg weight	male Sprague-Dawley rats	hydroxylase-positive neurons attenuation loss caused by 6-hydroxydopamine-induced dopamine depletion	CBr2 activation CBr1 blocking	[121]
		2 mg/kg weight	CB2−/− mice	preservation of tyrosine hydroxylase–positive neurons	CBr1 antagonism	[121]
		not listed	SH-SY5Y human neuroblastoma cells	neuroprotective effects	CBr1 independent	[122]
	Nabilone	30 mg/kg	7 human patients	reduction of levodopa-induced dyskinesia	not listed	[123]
Alzheimer’s disease	CBD	10^−7^–10^−5^ M	PC12 cells	inhibition of Tau protein hyperphosphorylation	not listed	[124]
		20 mg/kg	male AβPPSwe/PS1ΔE9 (AβPP × PS1) mice	social recognition strengthening protection against neuroinflammation	not listed	[125]
		0.75 mg/kg	male AβPP/PS1 104 mice	neuroprotective effects	not listed	[126]
	THC	0.75 mg/kg	male AβPP/PS1 104 mice	neuroprotective effects	not listed	[126]
		1.5 mg/3× day	54 human patients	no significant effect on dementia-related neuropsychiatric symptoms compared to placebo	not listed	[127]
Alzheimer’s disease		0.75 mg and 1.5 mg/2× day	22 human patients	no reduction of dementia-related neuropsychiatric symptoms well tolerated by vulnerable patients	not listed	[128]
	THC + CBD	0.75 mg/kg+ 0.75 mg/kg	Male AβPP/PS1 104 mice	the combination of THC and CBD exhibits a better therapeutic profile than single treatment	not listed	[126]
	Nabilone	0.5 mg/2× day	75-years old man (case report)	prompt reduction of agitation and resistiveness improvement of aggressive behavior	not listed	[129]
	CBD loaded nano-chitosan	not listed	Wistar rats	revealing of positive behavioral changes reducing Aβ plaques improving learning and memory	CBr1 and CBr2 increase	[102]
Multiple sclerosis	CBD	1, 5 or 10 µM	MOG35-55-specific T-cells	inhibition of the proliferation of encephalitogenic T cells	CBr1 and CBr2 independent	[130]
		5 mg/kg weight	C57BL/6 mice	amelioration of autoimmune encephalomyelitis clinical signs delay of disease progression	CBr1 and CBr2 independent	[130]
		5 mg/kg weight	TMEV-IDD-susceptible female SJL/J mice	improvement of motor deficits in the chronic phase of TMEV-induced demyelinization	adenosine A2A receptors	[53]
	Sativex	5 mg and 10 mg/kg weight	adult ABH mice	reduction of spasticity	not listed	[131]
		maximal 12 sprays/day	106 human patients	improvement of resistant spasticity	not listed	[132]
		8–10 sprays/day	30 human patients	reduction of spasticity	not listed	[133]
	Nabiximol	not listed	33 human patients	immunomodulatory activity	not listed	[134]
Glioblastoma	CBD	100 mM	U87 glioma cells	cell migration inhibition	CBr1 and CBr2 independent	[135]
		0.1–100 μmol/L	T98G, U87MG, and GL261	dose-dependent reduction of cell number enhancement the effect of irradiation	not listed	[136]
		5–50 µM	U87	dose-dependent induction of calcium influx time- and dose-dependent decrease of cell viability	TRPV2 activation	[137]
		2 mg/kg	female C57BL/6 mice	prime glioma cells to respond better to ionizing radiation	not listed	[136]
Glioblastoma	CBD lipid nanocapsules	200 μL	hCMEC/D3 cells	the enhancement of brain targeting	not listed	[104]
		2.5 mg and 5 mg/mL	U373	3.4-fold enhancement of in vitro glioma targeting in comparison with their equally-size empty controls reduction of the IC50 values	not listed	[100]
		150 μL	male ICR mice	the highest brain-targeting ability achieved with the smallest-sized nanocapsules	not listed	[104]
	THC	0.1–100 μmol/L	T98G, U87MG, and GL261	dose-dependent reduction of cell number irradiation enhancement	not listed	[136]
		2 mg/kg	female C57BL/6 mice	prime glioma cells to respond better to ionizing radiation	not listed	[136]
		various concentrations	U87, A172, SW1783, U373, T98G, SW1088, and LN405	dose-dependent reduction in cell viability, combined with temozolomide production of ROS in cancer cells	CBr1 and CBr2 dependent	[138]
		15 mg/kg	nude mice	decrease in tumor growth with temozolomide higher effect than using single compounds	CBr1 and CBr2 dependent	[138]

In contrast, neurodegenerative disorders have features other than those of tumors. Neurodegenerative disorders are primarily characterized by massive neuron loss [139]. In Alzheimer’s disease, cholinergic neurons massively die [140]. Parkinson’s and Huntington diseases are characterized by the loss of dopaminergic neurons, predominantly in the substantia nigra and globus pallidus, respectively [141]. The ECS is also involved in the development of neurodegenerative disorders. Alterations of the ECS are often difficult to interpret owing to the number of endocannabinoid mediators involved and the multifaceted nature of the changes. The changes in the ECS during these diseases may rather be described as system dysregulation, because its actions depend on the location and timing of the production [142]. β-Caryophyllene (BCP) is an artificial cannabinoid, a selective agonist of the CBr2 receptor that is not only expressed in the CNS but also in the immune system, and lacks psychoactivity. CBr2 agonism is associated with several anti-inflammatory mechanisms, including neurodegenerative pathways [143]. The results of the authors confirmed the suitability of BCP nanoparticles for nasal administration with enhanced absorption potential due to their slightly acidic pH and hypotonicity. BCP increases absorption owing to its lipophilic features [143]. There are many reviews discussing the potential of cannabinoid action in the brain, as well as nanoparticles crossing the BBB; however, none of them deals with targeted delivery into brain sites affected by neurodegenerative disorders. On the other hand, when talking about neurodegenerative disorders, the massive loss of neurons may occur in different brain areas and it is not predictable which ones will be attacked. Therefore, there is no need to have targeted delivery systems when the aim of the treatment is to support mature neurons, and/or proliferative active cells in neurogenic areas, such as hippocampus. Postnatal neurogenesis is an interesting topic to discussion. It occurs in neurogenic areas, such as the hippocampus, where cell proliferation occurs during the adulthood [144]. Neurodegenerative disorders are characterized by decreased hippocampal neurogenesis [145]. However, the literature on this topic is scarce.

## 4. Conclusions

The growing acceptance of *Cannabis* and cannabinoids has led to an increasing number of clinical trials testing various nanoproducts. CNS disorders are key therapeutic targets for cannabinoids, and nanoformulation platforms for cannabinoid nanoconjugates provide efficient transport across the BBB. Selected examples of cannabinoid nanoconjugates have shown enhanced bioavailability and improved bio-efficacy with promising outcomes in biomedical applications. We expect to see many results from clinical trials in the near future to evaluate the adverse effects and effectiveness of this treatment. However, precise delivery of these nanoformulations to pathological sites inside the brain remains a challenge.

## Data Availability

Not applicable.
